# Speech recognition can help evaluate shared decision making and predict medication adherence in primary care setting

**DOI:** 10.1371/journal.pone.0271884

**Published:** 2022-08-04

**Authors:** Maxim Topaz, Maryam Zolnoori, Allison A. Norful, Alexis Perrier, Zoran Kostic, Maureen George

**Affiliations:** 1 School of Nursing and Data Science Institute, Columbia University, New York, New York, United States of America; 2 Visiting Nurse Service of New York, New York, New York, United States of America; 3 Irving Institute for Clinical and Translational Research, Columbia University, New York, New York, United States of America; 4 School of Nursing, Columbia University, New York, New York, United States of America; 5 Department of Electrical engineering, Columbia University, New York, New York, United States of America; Mae Fah Luang University, THAILAND

## Abstract

**Objective:**

Asthma is a common chronic illness affecting 19 million US adults. Inhaled corticosteroids are a safe and effective treatment for asthma, yet, medication adherence among patients remains poor. Shared decision-making, a patient activation strategy, can improve patient adherence to inhaled corticosteroids. This study aimed to explore whether audio-recorded patient-primary care provider encounters can be used to: 1. Evaluate the level of patient-perceived shared decision-making during the encounter, and 2. Predict levels of patient’s inhaled corticosteroid adherence.

**Materials and methods:**

Shared decision-making and inhaled corticosteroid adherence were assessed using the SDM Questionnaire-9 and the Medication Adherence Report Scale for Asthma (MARS-A). Speech-to-text algorithms were used to automatically transcribe 80 audio-recorded encounters between primary care providers and asthmatic patients. Machine learning algorithms (Naive Bayes, Support Vector Machines, Decision Tree) were applied to achieve the study’s predictive goals.

**Results:**

The accuracy of automated speech-to-text transcription was relatively high (ROUGE F-score = .9). Machine learning algorithms achieved good predictive performance for shared decision-making (the highest F-score = .88 for the Naive Bayes) and inhaled corticosteroid adherence (the highest F-score = .87 for the Support Vector Machines).

**Discussion:**

This was the first study that trained machine learning algorithms on a dataset of audio-recorded patient-primary care provider encounters to successfully evaluate the quality of SDM and predict patient inhaled corticosteroid adherence.

**Conclusion:**

Machine learning approaches can help primary care providers identify patients at risk for poor medication adherence and evaluate the quality of care by measuring levels of shared decision-making. Further work should explore the replicability of our results in larger samples and additional health domains.

## Background and significance

### Introduction

Asthma is one of the most common adult chronic illnesses affecting 19 million adults (7.7%) in the United States [1]. Relative to white (8.1%) and Hispanic adults (5.8%), black adults have higher asthma prevalence (9.2%), [1] higher death rates (22.7 v. 8.1 v. 7.1/million) [1] and are about three times more likely to have severe asthma [2]. Inhaled corticosteroids (ICS) are a safe and effective treatment for uncontrolled asthma and, if used regularly, could prevent nearly every asthma-related hospitalization and death [3]. Yet, ICS adherence is poor, with black adults having lower adherence than Hispanics or whites [4].

Recent studies show that shared decision-making (SDM), a patient activation strategy that engages patients and their providers in treatment decisions, improves clinical outcomes in asthma, [5] including improved patient adherence with ICS. In SDM, the health provider’s role is to facilitate discussion of the risks and merits associated with specific treatment options in the context of patients’ goals and preferences and in a manner that activates patients to engage in self-management [6]. During SDM, health providers jointly consider treatment options with their patients to reconcile differences and reach mutually agreed-upon decisions that align patients’ needs and evidence-based guidelines [7].

However, implementing SDM is challenging due to several reasons. First, although patient engagement is a critical component of SDM, it is hard for health providers to evaluate the quality of patient-perceived SDM during health encounters due to barriers such as power imbalance between providers and patients [8, 9]. Second, while health providers are supportive of SDM during care delivery, there are barriers to SDM in real-world clinical settings, including lack of time and limited health provider knowledge of the SDM approaches. Prompt recognition and support of increased SDM between patients and health providers may increase the occurrence of SDM conversations and, in turn, increase medication adherence [10]. Asthma patients with poor ICS adherence should be prioritized for SDM interventions given existing evidence demonstrating the potential for improved asthma outcomes [10–12]. However, estimating patients’ risk for poor ICS adherence is not widely applied and remains challenging [13, 14].

### Related work

Recent technological and artificial intelligence advances can help resolve some of these pressing challenges. For example, computational methods can derive human behavioral signals from speech and language [15]. These computational methods often rely on acoustic and linguistic features extracted from audio-recorded conversations. Acoustics features reflect the shape and amplitude of the acoustic waveform, often including the rhythmic structure of speech frequency and spectral domain [16]. On the other hand, linguistic features refer to the content of the recorded conversation and commonly include characteristics such as the complexity of the grammar and syntax [17].

Several studies used recorded encounters between patients and health providers to predict other health outcomes. For example, one study applied machine learning to successfully predict the presence of post-traumatic stress disorder based on recorded conversations (using mostly acoustic features) between psychologists and army veterans [18]. Another study used transcribed interviews with psychiatric patients to predict patients’ psychosis onset [19]. An additional recent study used audio-recorded military couples’ conversations to predict suicide risk [20]. This study used a combination of acoustic and linguistic features to achieve risk prediction goals. A recent literature review presents a summary of the successful development of diagnostic and screening algorithms built on patients’ spoken language for detecting mental disorders [16]. Speech analysis and natural language processing methods and techniques have also been widely used to detect neurological disorders like Alzheimer’s and dementia. A recent systematic approach to Alzheimer’s disease detection from speech and language showed that a combination of acoustic and linguistic features could provide promising results in detecting Alzheimer’s and dementia from audio-recorded patient spontaneous speech [17].

### Study contribution

Our extensive literature search did not identify previous studies applying similar methods to asthma or medication adherence domains. This study extended and validated the emerging evidence of the effectiveness of speech and language technologies. Our major contribution is applying machine learning on audio recorded data to automatically identify care quality and predict patient outcomes in primary care settings.

### Study aims

Specifically, this study aimed to explore whether machine learning algorithms built on audio-recorded patient-primary care provider (PCP) encounters can be used to: 1. Evaluate the level of SDM during the encounter, and 2. Predict self-reported levels of patient’s ICS adherence (up to 3 months after the encounter).

## Methods

**Fig 1:**
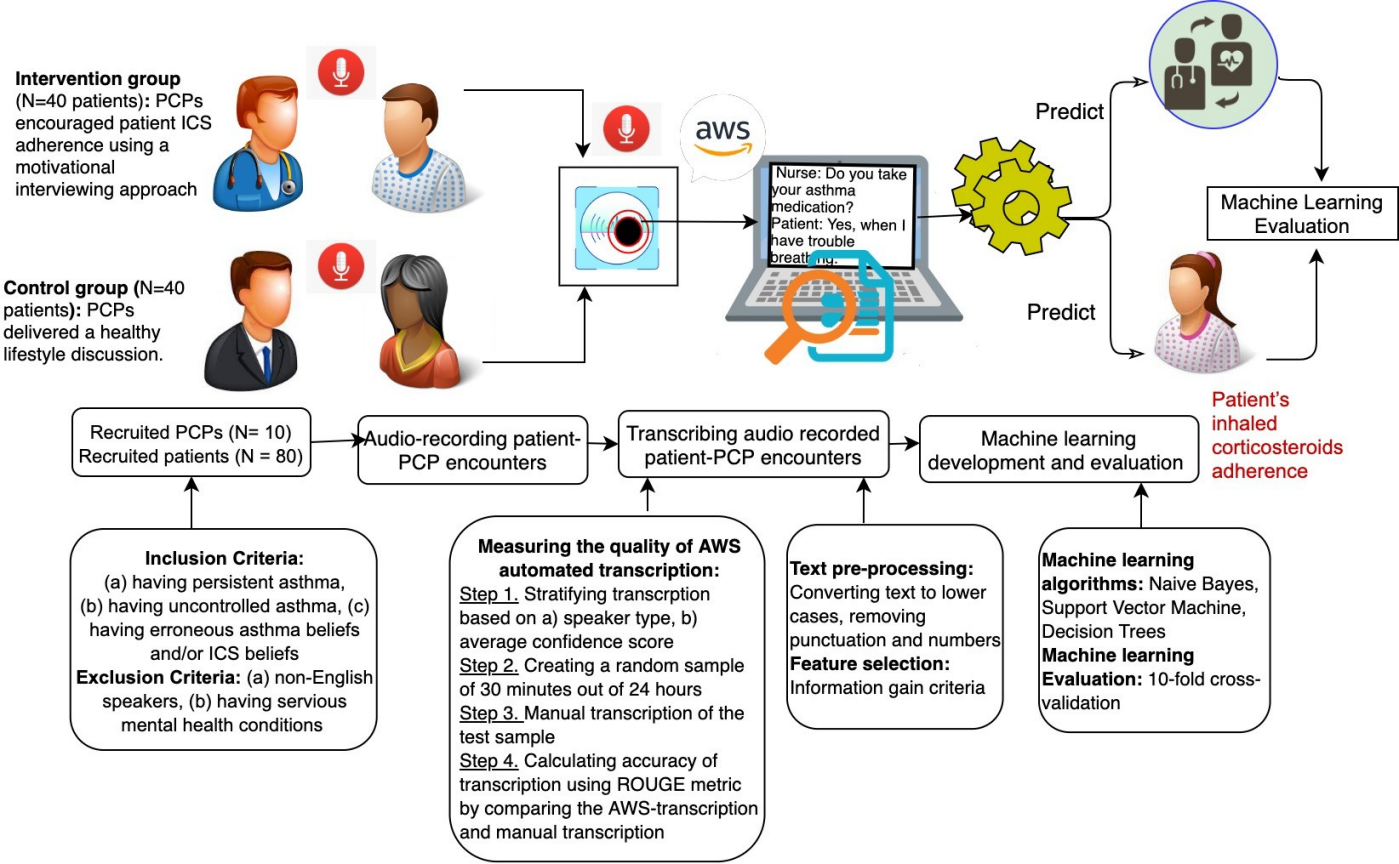
Study methods overview.

This study used secondary data from a study aimed at evaluating the impact of an SDM-based intervention on asthma control [21]. Next, we briefly describe the original study and then provide methodological details about the current study. Fig 1 shows an overview of the study’s methodology.

### Original study

#### Study settings

This study was conducted in two federally qualified health centers in Philadelphia (PA), serving areas with the highest asthma prevalence in the city. In the study, PCPs (primary care physicians or nurse practitioners) addressed erroneous asthma and medication beliefs in 80 patients (mean age 45; 83% female; 91% black/African American; 9% multiracial) with uncontrolled asthma using a standardized SDM-based intervention. The study was approved by Columbia University and the University of Pennsylvania’s institutional review boards. The full protocol has been reported elsewhere [21].

*Inclusion criteria*. Patients were included in the study if they met the following criteria: (a) had persistent asthma defined as PCP-diagnosed asthma requiring ICS; (b) had uncontrolled asthma based on the Asthma Control Questionnaire [22]; and (c) had erroneous asthma beliefs and/or ICS beliefs measured by the Conventional and Alternative Management for Asthma instrument [23].

*Exclusion criteria*. Patients were excluded if they were (a) non-English speakers or (b) had severe mental health conditions.

#### Study design

In this group-randomized trial, ten providers were randomized into the active SDM intervention condition (n = 5) or a dose-matched attention control condition (n = 5). Providers then delivered the active or control condition to 80 adult patients meeting inclusion and exclusion criteria (40 patients in each group).

*Intervention group*. In the intervention group, PCPs were instructed to encourage patient ICS adherence using a 9-minute motivational interviewing approach that advances SDM using the following key principles: (a) raise the subject of non-adherence to the medication; (b) provide feedback; (c) enhance the patient’s motivation using motivational interviewing techniques, (d) and advise and negotiate treatment options with the patient. Motivational interviewing is a patient-centered counseling approach engaging patients in a collaborative partnership with clinicians to change patients’ behaviors [24].

*Control group*. PCPs delivered a 9-minute healthy lifestyle discussion in the control group. There were no restrictions on what providers could discuss during the visit in the control group.

#### Study outcomes

The study used two validated tools to assess outcomes:

1. *Patient-reported level of SDM*: All patients completed the "SDM Questionnaire-9" (SDM-Q-9) [25]. SDM-Q-9 is a 9‐item, 6‐point Likert scale instrument (range: 1 completely disagree- 6 completely agree) to evaluate patients’ perspectives of the degree to which a PCP uses SDM during healthcare encounters. We selected this questionnaire because a recent systematic review led by our team found SDM-Q-9 to be superior in quality to other SDM instruments, especially when capturing SDM dimensions in chronic care visits [26]. Example questions are, "My doctor made clear that a decision needs to be made" and, "My doctor asked me which treatment option I prefer." Higher scores indicate greater levels of shared decision-making. The patients completed SDM-Q‐9 immediately after the patient participant-PCP encounter.
2. *Medication Adherence Report Scale for Asthma (MARS-A)* *[27]*: All patients completed the MARS-A to measure medication adherence. MARS-A is a 10-item, self-report measure of adherence with ICS. Example questions are "I only use it [ICS] when I need it" and "I try to avoid using it [ICS]." Each question is scored on a scale between 1 to 5 (range: 1 always- 5 never, summed and divided by 10 to give a range 1–5); a score of 4.5 or higher indicates greater adherence and correlates with objective measures of adherence as well as prescription refills. The patient completed the MARS-A four times: 1) Immediately before the patient participant-PCP encounter; 2) After one month; 3) After two months; and 4) After three months. The four measurements were implemented to understand longitudinal patient ICS adherence trajectories.

While the original study analysis is under review, see more details about the study protocol [21] and preliminary findings described elsewhere [28].

### Current study

The methods for the present study are divided into 2 phases, as described below:

#### Phase 1: Processing the audio-recorded verbal communication of PCP-patient encounters

All verbal communications during patient-PCP encounters were audio-recorded in both control and intervention groups. To process the audio-recorded patient-PCP encounters, we used the following steps:

*Step 1*: *Voice-to-text transcription*: We used the Amazon Web Service (AWS) Amazon Transcribe to transcribe all audio-recorded PCP-patient encounters (n = 80) to text. Amazon Transcribe is a speech-to-text Health Insurance Portability and Accountability Act of 1996 (HIPAA) compliant service that automatically creates text transcripts from audio files. Overall, our total recordings (both control and intervention groups) length was about 24 hours, with average encounter recording lasting about 18 minutes.

*Step 2*: *Measuring the quality of automated transcription*: To measure the quality of the transcript provided by the Amazon Transcribe, we used the following approach:

Creating a test sample: We evaluated the Quality of automated transcription of AWS by taking a random sample (2%, ~30 minutes out of 24 hours) of all the transcriptions. To create this random sample, we first stratified the transcription based on a) Speaker type, 50% recordings of PCPs, and 50% of patient participants. Amazon Transcribe includes a feature to label each fragment with the identified speaker. b) The average confidence score of transcription for each encounter. The Amazon Transcribe created the confidence score automatically (ranging from 1–100, with higher numbers indicating higher confidence scores) and showed how confident the system was with the transcription. The average Amazon Transcribe confidence score was .88 (minimum = .77, maximum = .98). We extracted 10 minutes of the evaluation sample from the transcribed segments with the lowest Amazon Transcribe confidence scores (range .77-.86); 10 additional minutes of the evaluation sample from the transcribed segment with medium Amazon Transcribe confidence scores (range .87-.9); and ten additional minutes of the evaluation sample from the transcribed segment with highest Amazon Transcribe confidence scores (range .91-.98).

Manual transcription of the test sample: To evaluate the quality of automated transcription by Amazon Transcribe, a member of our team transcribed all the recorded speech and compared them with the Amazon Transcribe’s transcription.

Step 3- Quantifying the transcription quality using the ROUGE technique: each evaluation segment was manually transcribed. Transcription quality was quantified using the Recall-Oriented Understudy for Gisting Evaluation (ROUGE) metric [29]. To evaluate the quality of automated transcription by Amazon Transcribe. To calculate the ROUGE score, human-transcribed segments are compared with speech-to-text generated text segments, producing scores reflective of transcription accuracy. We calculated ROUGE recall (how much of the human-transcribed text is captured by the automated speech-to-text transcription), ROUGE precision (how much of the automated speech-to-text transcription was accurate compared to human-transcribed text), and ROUGE F-score (harmonic mean between precision and recall).

#### Phase 2-Utilizing machine learning algorithms to evaluate the level of SDM and predict ICS adherence

*Study outcomes*. For machine learning algorithms, we dichotomized the score of SDM-Q-9 into either a low-moderate level of SDM (average SDM score <5) or a high level of SDM (average SDM score > = 5) [25]. Because MARS-A scores collected at four different time points were consistent within patients (more than 80% internal consistency), we also created an additional average MARS-A score across the four-time points. We dichotomized the average MARS-A score into either low self-reported adherence (average MARS-A score <4.5) or high self-reported adherence (> = 4.5). Previous studies recommended this cut-off as it is correlated with higher prescription refill rates and objective measures (electronic monitoring) of ICS use [30].

*Study dataset and data pre-processing*. The study dataset included all the audio recorded of verbal communication of patient-PCP encounters that were transcribed by the Amazon Transcribe to text. Each recorded encounter was linked to the score of the SDM-Q-9 and the score of the MARS-A completed by the patients. To prepare the transcribed encounters for machine learning, we implemented several text pre-processing steps, including removing punctuation, converting all letters to lower-case, and removing numbers. We also used a stemming technique that reduces words to their roots, e,g. by removing suffixes such as "*ing*.*"*

*Features used in machine learning models*. Overall, after pre-processing, the data included 11,348 features representing unique words and expressions that appeared in transcribed patient-PCP encounters. All data on these features were complete since they were represented as either present or absent in a specific patient-PCP encounter.

*Machine learning algorithms*. We implemented a series of machine learning algorithms to estimate the level of SDM and predict patients’ adherence to ICS. We applied three algorithms [31], including: 1. Naive Bayes- a probabilistic classifier based on the Bayes’ theorem that (naïvely) assumes independence between the predictor features; 2. Support Vector Machines- a supervised machine learning classifier that performs classification by finding a hyperplane that maximizes the margin between the two classes; and 3. J48 Decision Tree- a supervised classifier that uses a decision tree (as a predictive model) to go from features (represented in the branches) to classification conclusions (represented in the tree leaves).

The algorithms were implemented in Weka with default settings. More information about the algorithms and their operation mechanism for data classification is provided in S1 Appendix [31].

We also conducted feature selection, using information gain criteria [31], to reduce the sets of features to about 1,000 of the most informative features. These features were visualized using a word cloud technique where features with higher entropy scores (indicative of more informative features) were represented as words of larger sizes.

*Measuring the predictive performance of the algorithms*. Predictive performance of each machine learning algorithm was evaluated using standard metrics [31] of precision (defined as the number of true positives out of the total number of predicted positives), recall (defined as the number of true positives out of the actual number of positives) and F-score (defined as the weighted harmonic mean of the precision and recall). Because our sample was relatively small, we used 10-fold cross-validation to estimate the predictive performance. Cross-validation evaluates predictive algorithms by partitioning the original sample into a training set to train machine learning algorithms and a testing set to evaluate them [31]. In 10-fold cross-validation, this procedure is repeated ten times.

*Sensitivity analysis*. Finally, to account for the possible effect of the study intervention, we conducted sensitivity analysis by repeating each analysis for both experimental and control study groups. We compared algorithms’ predictive performances to explore whether results remained consistent regardless of the study group.

## Results

### Voice-to-text transcription

Overall, the transcription quality was relatively good with ROUGE scores of precision = .91, recall = .89, F-score = .9. No significant differences were found between ROUGE scores by speaker type (PCP versus patient participants).

### Study outcomes

In our sample, 30 participants (38%) reported high levels of SDM, while the rest reported low-medium levels of SDM. In addition, 22 participants (28%) were classified as having high ICS adherence, while the rest had low ICS adherence.

### Text mining

Table 1 shows that our machine learning algorithms achieved good predictive performance for SDM, with the best results produced by the Naive Bayes algorithm (precision, recall, and F-score = .88). In addition, machine learning algorithms achieved good predictive performance for predicting ICS adherence, with the best results produced by the Support Vector Machines algorithm (precision = .87, recall = .88, and F-score = .87). Sensitivity analysis for each study group (control and experimental) showed similar trends indicating good overall machine learning predictive performance.

**Table 1:** Machine learning predictive performance.

Outcome	Metric	Naive Bayes	Support Vector Machines	J48 Decision tree
**SDM**	Precision	0.88	0.85	0.72
	Recall	0.88	0.85	0.73
	F-score	**0.88**	0.85	0.71
**MARS-A total average**	Precision	0.82	0.87	0.71
	Precision	0.82	0.87	0.7
	Recall	0.83	**0.88**	0.7
**MARS-A initial**	F-score	0.81	0.87	0.7
	Recall	0.84	0.8	0.75
	F-score	**0.82**	0.79	0.74
**MARS-A after one month**	Precision	0.76	0.84	0.75
	Recall	0.75	0.78	0.75
	F-score	0.72	**0.75**	**0.75**
**MARS-A after two months**	Precision	0.72	0.72	0.63
	Recall	0.7	0.72	0.63
	F-score	0.7	**0.72**	0.63
**MARS-A after three months**	Precision	0.83	0.79	0.68
	Recall	0.83	0.79	0.68
	F-score	**0.83**	0.78	0.68

**Fig 2:**
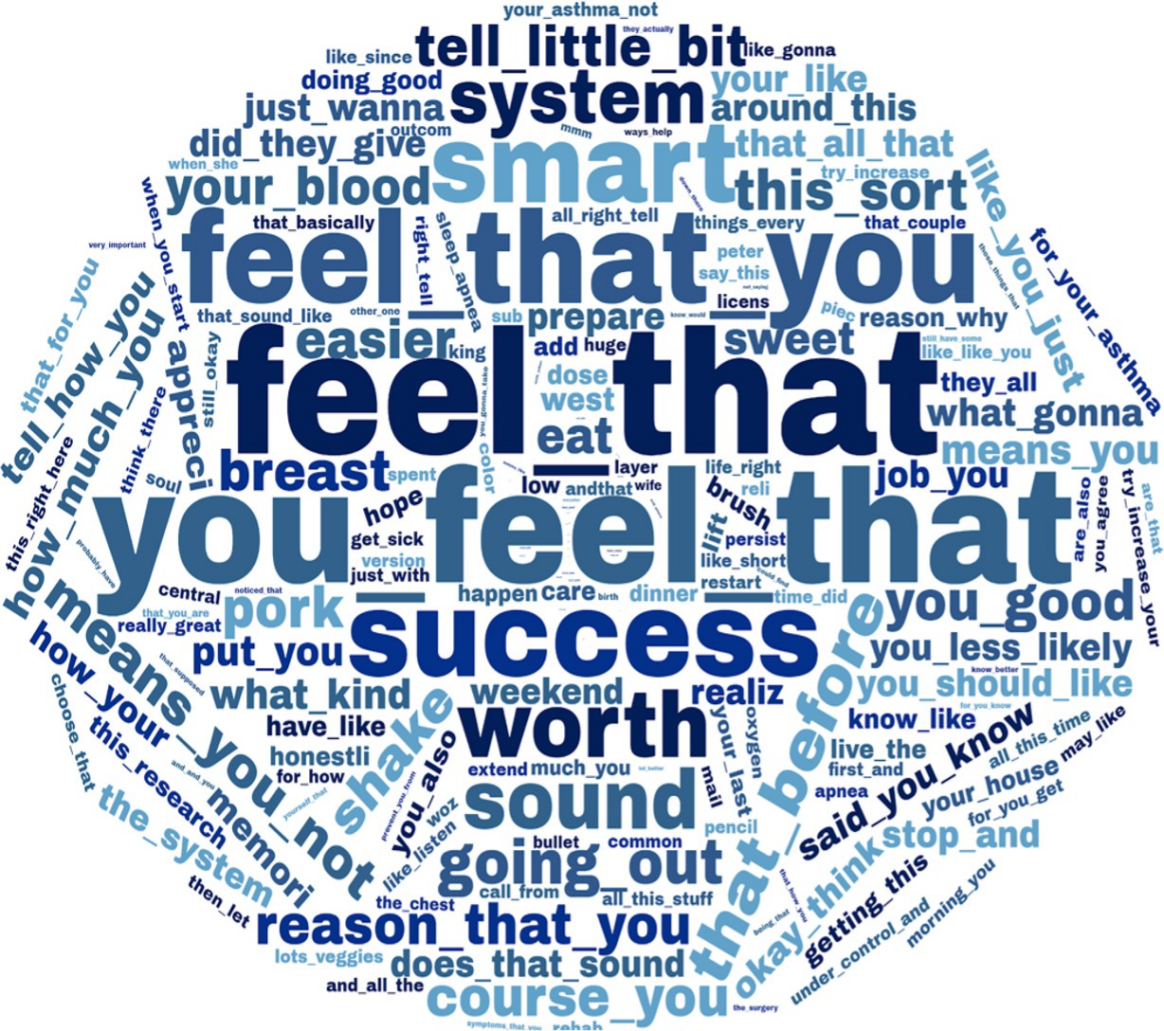
Words and expressions associated with higher levels of shared decision-making.

**Fig 3:**
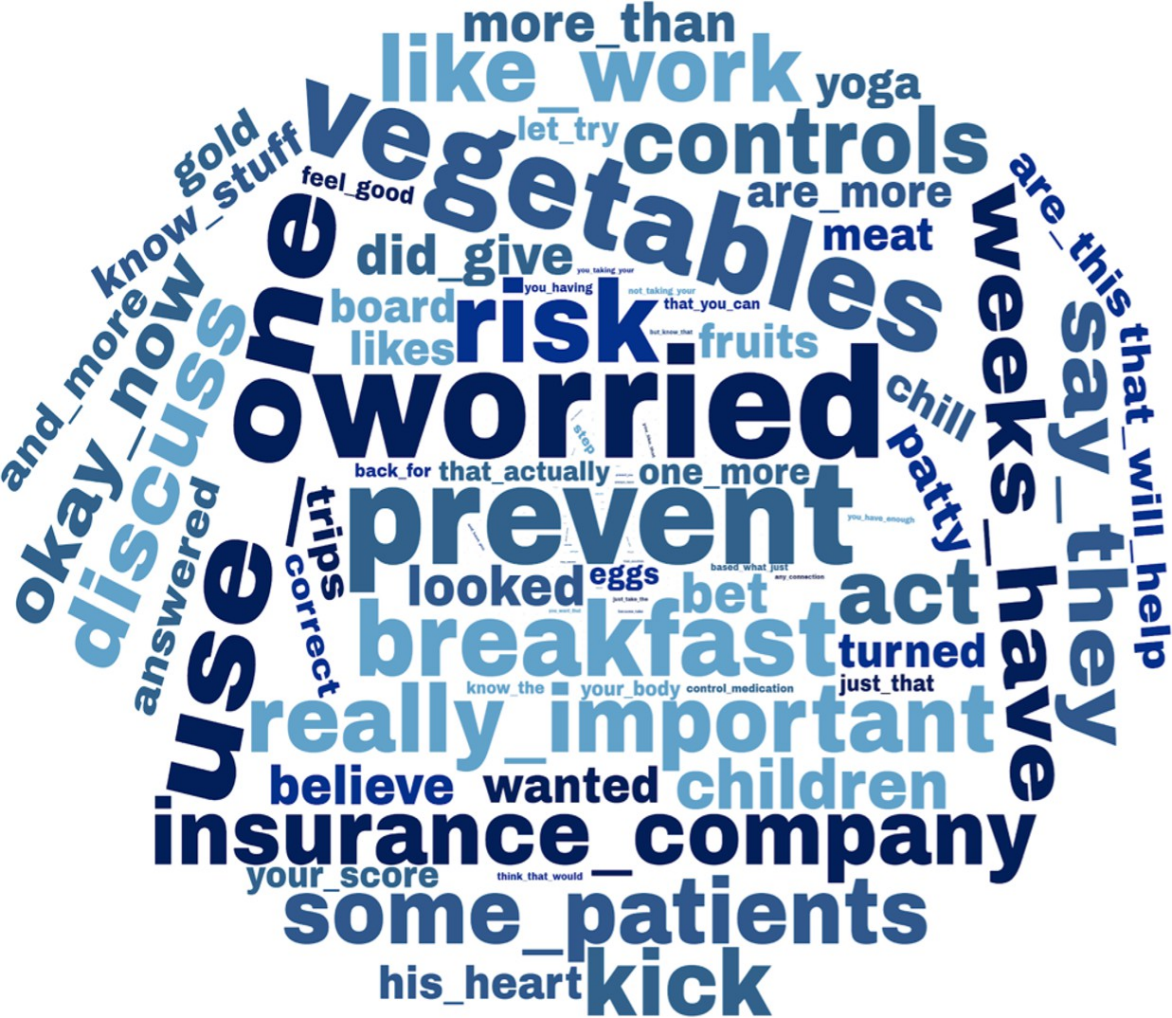
Words and expressions associated with poor Inhaled corticosteroids adherence.

Figs 2 and 3 depict the most informative features extracted by machine learning. For SDM, visual examination of Fig 2 suggests that words and expressions related to reflecting feelings, such as "feel that" or "you feel that," and positive expressions, such as "success" or "worth," were associated with higher SDM levels. For ICS adherence, Fig 3 suggests that concerning words and expressions, such as "worried," "risk," or "prevent," and language related to food, such as "breakfast," "vegetables," or "patty," were associated with lower levels of ICS adherence.

## Discussion

The results of this study provide preliminary evidence of the successful application of computational methods to derive human behavioral signals from speech and language [15]. Specifically, our results show that the content of audio-recorded patient participant-PCP conversations can be used to automatically evaluate perceived shared decision-making and predict patient outcomes.

Machine learning algorithms applied in this study were able to accurately detect patient-reported levels of SDM during patient participant-PCP encounters. There are widely documented benefits of increased SDM during patient-health provider encounters, including increased medication adherence, reduced asthma-related health care visits, and improved asthma control [26]. Automated detection of SDM during clinical visits may prompt the timely onset of SDM conversations and yield better patient outcomes. This is consistent with previous evidence that suggests that interventions targeting both the patient and provider have a greater positive effect on the occurrence of SDM when compared to patient-specific interventions alone [11]. Automated detection of SDM during patient-provider encounters would allow for the investigation of dyadic patient-provider interventions.

Further analysis of language associated with higher levels of patient-reported SDM identified several broad categories of words and expressions. For example, language reflecting feelings (e.g., "feel that" or "you feel that") and positive language (e.g., "success" or "worth") was associated with higher levels of SDM. These findings are well aligned with previous literature that theoretically defines the dimensions of SDM in clinical care [9]. In addition, core principles of motivational interviewing aimed at improved SDM suggest that clinicians should "Express empathy through reflective listening" and "Support self-efficacy and optimism" [32]. As guided by similar principles, motivational interviewing (with core elements of engaging, evoking, motivating, and planning) targets behavior change, while SDM incorporates discussion for reasonable options to make such decisions. Using machine learning algorithms that measure motivational interviewing quality may further support SDM implementation during patient-health provider encounters.

Machine learning algorithms applied in this study also showed a promising trend of accurately identifying patients at risk for poor ICS adherence. Moreover, algorithms’ predictive performance remained relatively high for predicting longitudinal risks of poor ICS adherence (up to 3 months). In the near future, such algorithms have the potential to be adopted into clinical practice and integrated into everyday care to identify patients at risk for poor ICS adherence. These high-risk patients can be prioritized for SDM interventions to increase their ICS adherence.

When analyzing language associated with poor ICS adherence, we found one common category that included words and expressions indicating concern (e.g., "worried," "risk," or "prevent"). We speculate that this language reflected PCPs’ patient adherence status concerns. These concerns might have been frequently justified; thus, we identified lower levels of ICS adherence among patients with such language.

In addition, language related to food (e.g., "breakfast" or "vegetables") indicated lower levels of ICS adherence. On the one hand, patient participant-PCP discussions in the study’s control group consisted of healthy lifestyle coaching, including nutritional recommendations. Therefore, although our subgroup sensitivity analysis showed similar predictive trends for each group, our results might have been skewed by the content of these conversations. On the other hand, we speculate that food-related discussions were prevalent when PCPs also had concerns regarding patients’ ICS adherence status. More research is needed to understand these trends further.

Finally, our evaluation of the accuracy of the automated speech-to-text transcription showed that roughly 90% of the patient participant-PCP encounter conversations were transcribed correctly (F-score = .9). A recent systematic review found that these results are consistent with other studies on the quality of medical speech-to-text transcription [33]. For example, one study found an error rate of 7% in the transcription of physician-generated clinical documents in hospitals [34]. On the one hand, this level of accuracy proved sufficient for implementing good-performing machine learning algorithms applied in this study. On the other hand, roughly one in ten words was transcribed incorrectly or missed by the automated system. Such a level of accuracy might not yet be suitable for clinical purposes requiring human use of the automatically generated transcripts. Given significant progress in the accuracy of speech-to-text algorithms in the last several years [33], we are hopeful that transcription accuracy will improve. Accuracy improvement will enable a broader range of clinically meaningful uses, such as an automated patient-health provider encounter summarization or intelligent conversational assistants that might help patients achieve better medication adherence.

### Limitations

This study has several significant limitations. First, our dataset was limited to 80 patient participant-PCP recorded encounters. This data is relatively small, and we cannot draw widely generalizable conclusions based on this sample. Second, we evaluated the quality of automated speech-to-text transcription in a relatively small sample; thus, our findings should be validated in further studies. Third, our analysis focused solely on features extracted from the content of audio-recorded encounters (transcribed by the Amazon Transcribe). In the next phase of this study, we plan to extract temporal vocal features (time-domain features), such as the energy of signal or maximum amplitude, and spectral features (frequency-based features), such as fundamental frequency and spectral centroid from the speech. These features may help improve the performance of machine learning algorithms in evaluating the level of the SDM during patient-PCP encounters and predicting ICS adherence. Fourth, some of the applied machine learning algorithms (e.g., Naive Bayes) are more prone to overfitting (i.e., producing artificially high predictive performance) with small-size datasets. Although we implemented 10-fold cross-validation to reduce the risk of overfitting and other algorithms with less risk of overfitting (e.g., Support Vector Machines) also showed good predictive results, our analysis should be verified with larger sample sizes.

#### Further work

In the next phase of this study, we plan to extract temporal vocal features (time-domain features), such as the energy of signal or maximum amplitude, and spectral features (frequency-based features), such as fundamental frequency and spectral centroid from the speech. These features may help improve the performance of machine learning algorithms in evaluating the level of the SDM during patient-PCP encounters and predicting ICS adherence. Further work should also explore the replicability of our results in larger data samples and additional health domains. Finally, in the near future, such machine learning approaches can be applied to create intelligent health assistants to help clinicians identify at-risk patients. Such intelligent assistants can also be applied to evaluate the quality of ongoing care and uncover which clinician verbal behaviors are associated with better patient-reported SDM.

## Conclusions

This study applied several machine learning methods to process audio-recorded healthcare encounters. The major contribution of this study is validating a hypothesis that audio recordings of patient participant-PCP encounters can be used to successfully evaluate the quality of SDM and predict patient ICS adherence.

## Supporting information

S1 Appendix (DOCX)
