# Supplementary material for: Speech recognition can help evaluate shared decision making and predict medication adherence in primary care setting
Source: PLoS One. 2022 Aug 4;17(8):e0271884. doi: 10.1371/journal.pone.0271884 (PMC9352008; doi:10.1371/journal.pone.0271884)
Supplement: S1 Appendix — (DOCX) [file pone.0271884.s001.docx]

**Appendix**

**Naïve Bayes**

Naïve Bayes is a conditional probability model that assumes the independence relationship between features. Given a problem instance to be classified, represented by a vector X = (Xi …. Xn) x=(x1,…,xn), it assigns to this instance probabilities:

P(C_k_ | X_1,_ …. X_n_)

Using [Bayes' theorem](https://en.wikipedia.org/wiki/Bayes%27_theorem), this conditional probability can be decomposed as:


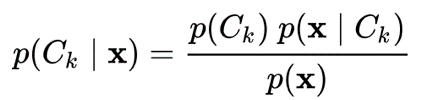


Using the formula above, we can get prediction of the outcomes of observations based on the following formula:


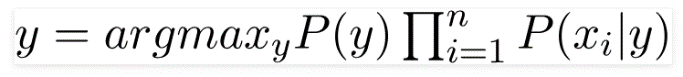


**Support vector machine**

# A support vector machine is a binary classifier that identify the maximum margin hyperplane that separate the two classes in the data. Suppose the sample of data for classification is denoted by X ∈ R^N*M^ (N is the participants in the sample and P is the number of features), and the data from participant i are denoted by X^(i)^ ∈ R^(M)^.

# Let assume that Y is the outcome variable denoted by Y^(i)^ ∈ {-1, 1}. Y presenting the class labels for all participants (where -1 and 1 are numerical labels for the two classes). The optimization problem to find the optimal hyperplane (described by weights W∈ R^(M)^ and intercept term b ∈ R is as follows:

Min_(w,b)_1/2 ||W||^2^,

subject to Y^(i)^ (W^T^ X^(i)^ + b) >= 1, i ∈ {1, …, N}.

When the optimal hyperplane [W_opt_, b_opt_] has been found, the predicted class label for the subject i is obtained as the sign of W^T^_opt_ X^(i)^ + b_opt_. This formulation assumes that the data are fully linearly separable. When this is not the case, slack variables and a tolerance parameter are generated to obtain hyperplanes that tolerate small misclassification errors.

**Decision Tree**

The algorithm uses entropy to calculate the homogeneity of sample. If the sample is completely homogeneous, the entropy is Zero. But if the sample is equally divided between two (n) classes of outcome variables, the entropy is one. To build the decision tree, we need to calculate entropy. Entropy is a measure of disorder or uncertainly and the goal of the decision tree or other machine learning algorithms is to reduce entropy.


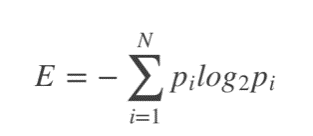


P_i_  is the probability of randomly selecting an example in class i.

To measure the reduction in our target class of data given additional information about it, we will use information gain formula, which is:


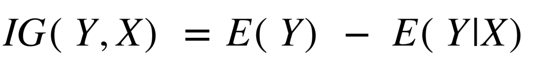


We simply subtract the entropy of Y given X from the entropy of Y to calculate the reduction of uncertainty about Y given additional information that X provides about Y. The greater reduction in the uncertainty, the more information is gained about Y from X.

Decision tree algorithms computes the information gain for each feature (variables) in the sample, to create branches of tree and do the analysis.
